# Comparison of two Bayesian methods to detect mode effects between paper-based and computerized adaptive assessments: a preliminary Monte Carlo study

**DOI:** 10.1186/1471-2288-12-124

**Published:** 2012-08-17

**Authors:** Barth B Riley, Adam C Carle

**Affiliations:** 1 Department of Health Systems Science, M/C 802, College of Nursing, University of Illinois at Chicago, 845 S. Damen Ave., Chicago, IL 60612, USA; 2 University of Cincinnati School of Medicine, Cincinnati Children's Hospital Medical Center, 3333 Burnet Avenue, MLC 7014, Cincinnati, OH 45229, USA

## Abstract

**Background:**

Computerized adaptive testing (CAT) is being applied to health outcome measures developed as paper-and-pencil (P&P) instruments. Differences in how respondents answer items administered by CAT vs. P&P can increase error in CAT-estimated measures if not identified and corrected.

**Method:**

Two methods for detecting item-level mode effects are proposed using Bayesian estimation of posterior distributions of item parameters: (1) a modified robust *Z* (*RZ*) test, and (2) 95% credible intervals (*CrI*) for the CAT-P&P difference in item difficulty. A simulation study was conducted under the following conditions: (1) data-generating model (one- vs. two-parameter IRT model); (2) moderate vs. large DIF sizes; (3) percentage of DIF items (10% vs. 30%), and (4) mean difference in *θ* estimates across modes of 0 vs. 1 logits. This resulted in a total of 16 conditions with 10 generated datasets per condition.

**Results:**

Both methods evidenced good to excellent false positive control, with *RZ* providing better control of false positives and with slightly higher power for *CrI*, irrespective of measurement model. False positives increased when items were very easy to endorse and when there with mode differences in mean trait level. True positives were predicted by CAT item usage, absolute item difficulty and item discrimination. *RZ* outperformed *CrI*, due to better control of false positive DIF.

**Conclusions:**

Whereas false positives were well controlled, particularly for *RZ*, power to detect DIF was suboptimal. Research is needed to examine the robustness of these methods under varying prior assumptions concerning the distribution of item and person parameters and when data fail to conform to prior assumptions. False identification of DIF when items were very easy to endorse is a problem warranting additional investigation.

## Background

Computerized adaptive testing (CAT) is widely used in education and has gained acceptance as a mode for administering health outcomes measures [1,2]. CAT offers several potential advantages over conventional (e.g., paper-and-pencil) administration, including automated scoring and storage of questionnaire data, and reduction of respondent burden. Instruments developed for paper-and-pencil administration frequently form the basis for CAT. In these situations, the transition to computerized adaptive testing requires establishing the equivalence between CAT-administered measures and their original paper-and-pencil version [3,4]. A meta-analytic review of 65 studies comparing computerized an paper-and-pencil administration of patient-reported outcome measures suggests that scores obtained by computer are comparable to those obtained by conventional modes of administration [4]. This study, however, did not focus on CAT. Unlike computer-based assessment, CAT selects items for administration based on item parameters that, if not accurate for CAT mode of administration, may diminish the reliability or efficiency of CAT [5,6]. Item-level mode effects, in other words, may have a greater effect on CAT compared to other assessment modalities. The shift in item parameters resulting from changes in administration mode reflects the presence of differential item functioning (DIF), which can be defined as differential performance (e.g., differences in level of endorsement) of an item between two or more groups matched on the total score or measure [7,8]. This paper will focus on the detection of DIF between CAT and paper-and-pencil administrations of a measure.

Methods used for assessing DIF by mode of administration fall into two general categories: (1) approaches based on classical test theory (CTT), such as comparisons of item *p* values, representing percentage of endorsement; and (2) methods based on item response theory (IRT) [9-12], including comparisons of item difficulty parameters. Confidence intervals of item endorsement probabilities (i.e., *p*-values) have been found to vary significantly by mode [13,14]. Pommerich [13] also presented the proportion of items statistically favoring each mode. In another study [15], item *p*-values and IRT item difficulty parameters were compared and scatterplots of item parameters across mode were constructed. Johnson and Green [16] compared *p*-values of items as well as conducted a qualitative examination of error types (e.g., transcription error, place value error, partial answer, computation error, misunderstanding) made by students in each mode. Keng, McClarty, and Davis [17] examined differences in mode at the item level by comparing *p*-values and differences in chosen response category and by computing IRT-based DIF tests. Finally, Kim and Huynh [18] employed a robust-*Z* statistic to determine whether differences in item parameters across mode were statistically significant.

Though these studies often employed multiple methods of assessing item comparability, systematic comparisons across methods were not conducted. Nevertheless, there is reason to believe that some methods, such as item *p*-values may not be appropriate when detecting mode effects involving CAT-administered items. That is, differences in item *p*-values may not be valid indicators of DIF if the samples completing each mode of assessment differ in mean level on the measure. Moreover, item *p*-values can be influenced by the selective administration of items that takes place during CAT. For instance, CAT typically selects items that have an approximate probability of endorsement of 50% (i.e., items tailored to the individual to provide maximum information). Therefore, comparing CAT vs. P&P item *p*-values would likely result in items erroneously flagged as exhibiting DIF.

Several methods have been developed that attempt to overcome the limitations of classical procedures for detecting mode effects. Most of these methods are based on item response theory and involve comparisons of item parameters after matching of respondents according to trait level. Achieving accurate identification of DIF based on an IRT framework requires precise estimates of item parameters and person measures and the use of an appropriate measurement model [6]. However, a limitation of IRT-based methods is that missing data (e.g., resulting from CAT administration) can reduce accuracy in parameter estimates and in DIF detection [19,20]. In their simulation study, Robitzsch and Rupp [19] observed that when the missing data rate was 30% and data were missing at random, mean bias (difference between true and observed differences in item difficulty between groups) was 0.60, nearly two standard deviations above average bias across all conditions. CAT can reduce the number of items administered by as much as 90%, depending upon the size and quality of the item bank and criteria for stopping the test [21-23]. Therefore, higher rates of bias would likely occur when examining DIF in CAT-administered items with these methods.

Given the uncertainty in trait and item parameters, some investigators have recommended methods to identify DIF based on Bayesian probability theory. Bayesian approaches use probability distributions to model uncertainty in model parameters. These probability distributions represent prior beliefs or assumptions concerning the nature of the data and the level of uncertainty regarding various parameters. For instance, an investigator may specify that item discrimination parameters adhere to a lognormal distribution with log mean of 0 and variance of 0.5. The prior (particularly the prior variance) reflects uncertainty about the values before observing the data. Conversely, the *posterior* distribution reflects updated knowledge about parameter values after observing the data. Bayesian approaches make inferences using the posterior distribution. Unlike frequentist statistics, Bayesian methods do not rely on asymptotic (large-sample) theory in order to obtain standard errors, making Bayesian methods particularly attractive when small samples or missing data are involved.

Two general methods of DIF detection employing Bayesian methods have been proposed. The first approach is the use of Bayesian procedures to directly estimate DIF magnitude such as the Mantel-Haenszel (MH) test [24]. Zwick and her associates [25-27] tested an empirical Bayes (EB) formulation of the MH test and demonstrated that EB results more closely approximated targeted DIF values (i.e., values used to simulate DIF in the item response data) compared to standard MH. The latter finding was particularly true for the relatively small (N=1,000 per group) sample size condition. Power ranged from 63.8 to 81.4% depending on sample size and mean difference in proficiency between groups. However, Zwick and Thayer [26] acknowledged that EB resulted in a higher Type I error rate (ranging from 10% to 20%) compared to conventional MH.

A second approach involves estimation of the posterior distribution of model parameters, which can be used in subsequent DIF analyses [28-31]. Wang, Bradlow, Wainer and Muller [31] examined DIF for a given item by producing separate item difficulty estimates for each group. Posterior distributions of the item difficulty parameter (*b*_iG1_ and *b*_iG2_ for groups 1 and 2, respectively) are computed, and from this a Bayesian *p* value representing the number of times (*b*_iF_ - *b*_iR_) >0 can be used as an indicator of DIF. This procedure provided more accurate results compared to standard MH DIF analysis, especially when items were very easy to endorse [31]. In a similar application [29] posterior distributions of proficiency measures were used in two nonparametric regression models (one with and one without group membership as a covariate) to compute posterior mean *p* values for the likelihood ratio based on the two models. Using Bonferroni-adjusted *p* values and a total sample size of 900 simulees, the investigators were able to obtain power of .90 to 1.00 and false-positive rates well below the set alpha level of .05.

Despite these promising results, none of the studies employing posterior distributions of item parameter estimates assessed DIF in CAT-administered items. Moreover, to our knowledge there has been no application of Bayesian methods to the assessment of DIF between non-CAT and CAT-administered assessments. Standard methods of assessing DIF can be problematic when comparing CAT- and P&P-administered data because of the confounding of CAT item selection, sample differences in trait level, and actual mode effects.

### Rationale of the Study

It is common practice to employ paper-based forms when validating and scaling an item bank for use in CAT. Thus, it is important to determine that the resulting item parameters are not influenced by mode DIF. As suggested earlier, current methods of assessing DIF may not be appropriate when comparing adaptively and non-adaptively administered items. One solution would be to administer the entire item bank via computer and conventional modes of administration and then employ standard methods of DIF assessment. Whereas this approach could be used with small item banks, it would be quite burdensome to respondents and likely require collecting data apart from standard assessment practice with very large item banks.

Other researchers have already faced this issue. For example, to reduce respondent burden, the Patient Reported Outcomes Information System (PROMIS) only administered the entire set of initially developed PROMIS item to a small set of individuals from the total PROMIS calibration sample. This has limited the PROMIS collective’s ability to address some key issues, similar to what we raise here. Thus, while the less technical approach is possible, we suspect the common problem of needing to reduce respondent burden will generally limit the application of the less technical approach, indicating the need for alternative approaches. One alternative approach, which we present in this paper, is to develop procedures appropriate for detecting mode DIF in CAT vs. non-CAT-administered items, enabling assessment of DIF using data collected as part of standard assessment.

The purpose of the present study was to develop and evaluate two approaches to assessing item-level mode effects employing a Bayesian framework. In the following sections we outline this framework and describe the design and results from a preliminary Monte Carlo simulation study. The procedures are described and evaluated with respect to false-positive (i.e., DIF is detected when not simulated) and true-positive (i.e., DIF is detected when simulated) detection rates under several study conditions. We then examine factors associated with true and false DIF identification.

Research Questions:

1. How well does each method detect item-level mode effects as indicated by ROC analysis, true positive and false positive rates? In the present study, true positives are defined as identification of items as exhibiting mode DIF when mode DIF is simulated, which is also referred to as correct DIF detection. Conversely, false positives refer to flagging of items as exhibiting DIF when DIF was not simulated, which is also referred to as incorrect DIF detection.

2. What factors influence correct (true positive) and incorrect (false positive) detection of item-level mode DIF using each procedure?

## Methods

The methods employed in this study will be presented in three main sections. First, we describe the development and underlying assumptions of two Bayesian methods for detecting item-level mode effects. Second, we describe the simulation study, including its design and data generation procedures. The third section outlines the analysis of the simulated data.

### A Bayesian procedure for detecting item-level mode effects

In the proposed model, analysis of mode effects involved a three-step process:

Step 1. Estimate *θ* using item response data pooled across administration modes (CAT and P&P). That is, *θ* is obtained using item parameters based on the combined CAT and P&P response data. This is to ensure that item parameters estimated in subsequent steps are on a common metric.

Step 2. Using *θ*_i_ obtained in Step 1, estimate the posterior distributions of mode-specific item parameters for subsequent comparison in step 3.

Step 3. Estimate DIF for each item common across modes by assessing the difference in the posterior distributions of the item parameters (i.e., between *β*_j_^CAT^ and *β*_j_^P&P^). In the present study we examined two approaches to making this comparison. The first approach involved calculating a variation of the robust Z statistic [32] as shown in equation 1:

(1) $$\text{Robust}\;Z_{j}=\frac{\text{Med}\left(\beta_{j}^{\text{CAT}}-\beta_{j}^{P\&P}\right)}{0.74\left(IQR\left[\beta_{j}^{\text{CAT}}-\beta_{j}^{P\&P}\right]\right)}$$

where *Med* is the median and *IQR* is the interquartile range. The standard robust *Z* is asymptotically consistent with a standard normal distribution while minimizing the effect of extreme values. It has been used as a screening method for identifying stable items for IRT linking and DIF procedures [18,32]. Unlike previous application of the robust *Z* in which the median and interquartile range are based on point estimates of parameters for all items in the instrument, here these values are based on the posterior distribution of the parameters for item *j* in each administration mode.

The second approach involved constructing the 95% credible interval (*CrI*) of the CAT vs. P&P difference for item j’s difficulty parameter. This interval is computed by obtaining the 2.5 and 97.5 percentiles of item j’s posterior distribution of β_j_^CAT^ – β_j_^P&P^. In order to obtain a single value reflecting the level of mode DIF, we also computed the minimum difference of each bound of the *CrI* from zero (referred to as Δ *CrI*). Note that Δ *CrI* = 0 if the credible interval includes zero. The following priors were used in the model:

*θ*_i_ ~ *Normal*(0,1)

*α*_j_ ~ *Lognormal*(0,0.5)

*β*_*j*_*~ Normal(0,2)*

*Y*_ij_ ~ *Bernouli(P[Y*_*ij*_*= 1|θ, α, β])*

where the first value in parentheses for priors of *θ*_i_*α*_*j*_, and *β*_*j*_ is the prior mean and the second is the prior variance. These priors may be regarded as “semi-informative.” They are similar to priors employed in earlier IRT studies, with the exception that we selected a lognormal rather than a truncated normal prior for the discrimination parameters [33-35].

The Markov chain Monte Carlo estimation consisted of three parallel chains each with a separate and randomly generated set of starting values for model parameters. For each chain, the first 1,000 MCMC iterations were discarded (burn-in phase), followed by 500 iterations per chain retained for subsequent analysis. The total number of iterations and the length of the burn-in phase were chosen on the basis of preliminary examination of trace plots of item and person parameters which revealed good convergence of the three chains of parameter estimates (analysis results are available upon request). Using additional iterations or a longer burn-in did not change DIF analysis results.

### Simulation study

A preliminary Monte Carlo simulation study was performed to assess the accuracy of the proposed method for detecting item-level mode effects. Two interests guided the design and implementation of this simulation: First, in this preliminary study we decided to restrict our focus to uniform DIF instead of or in addition to non-uniform (i.e., discrimination) DIF. Second, we focused on instruments fitting a one- (1PL) or two-parameter (2PL) IRT model [9-11], which are commonly applied to health outcome measures. . Under the two-parameter (2PL) model, let *i* index respondents ( *i* = 1… *N*) and let *j* index items ( *j* = 1… *L*). The probability of respondent *i* endorsing item *j* can be expressed as

(2) $$P\left(Y_{\mathrm{ij}}=1|\alpha_{j},\beta_{j},\theta_{i}\right)=\frac{\exp\left(D\alpha_{j}\left[\theta_{i}-\beta_{j}\right]\right)}{1+\exp\left(D\alpha_{j}\left[\theta_{i}-\beta_{j}\right]\right)}$$

where *Y*_ij_ is the response to item *j* by respondent *iα*_*j*_ is the discrimination parameter and *β*_j_ is the difficulty parameter for item *jθ*_i_ is respondent *i*’s measure on the latent trait, and *D* is a scaling constant. In our simulations, *D* = 1.702 which makes the estimated response probabilities consistent with the normal ogive model and is used by the IRT estimation software employed in the study. In the one-parameter (1PL) case, all *a*_*i*_ are equal across items.

#### Study Design

In this study, the following factors were investigated: (1) data-generating model (one-parameter [1PL] vs. two-parameter [2PL] logistic IRT model); (2) DIF magnitude (|*β*^CAT^ – *β*^P&P^|) of 0.42 vs. 0.63 logits, which corresponds to “B” and “C” class DIF, respectively, according to Educational Testing Services criteria [25]; (3) DIF percentage (10% vs. 30% of items in the item bank), and (4) mean difference in *θ* estimates across modes of 0 vs. 1 logits. We employed a fully crossed research design that resulted in a total of 16 conditions, with 10 replications (datasets) per condition.

#### Data Generation

Data were generated for the present study in three steps: (1) generation of validation (paper-and-pencil) data, (2) generation of CAT item response data, and (3) CAT simulation, which produced item response datasets containing only those items selected by the CAT. Each of these steps is outlined in the following sections.

#### Generation of the Validation (Paper-and-Pencil) Item Parameters and Response Data

For each IRT model, a set of item parameters and corresponding item response datasets were generated. Both item banks consisted of 100 items. In the 1PL model, discrimination (*α*_j_) parameters for all items were set to 1.0; in the 2PL item bank, *α*_j_ parameters were randomly generated from a lognormal distribution with log mean = 0 and SD = 0.5, with values restricted to a range of 0.5 to 2.5. Discrimination parameters were limited to this range because items with very low discrimination (i.e., less than 0.5) are rarely used in item banks, whereas highly discriminating items (i.e., true discrimination parameters greater than 2.5) tend to be poorly estimated (i.e., positively biased) parameters [36]. For both item banks, item difficulty (*β*_*j*_) parameters were generated from a uniform distribution ranging from −3.0 to 3.0 logits, in increments of 0.25 logits. Person measures (*θ*_*i*_) for 500 simulees were generated using an *N*(0, 1) standard normal distribution.

#### Parameter Estimation

The generated item-response data were then used to estimate IRT item parameters (see Additional file 1). For both datasets, the standard deviation of the theta estimates was set to 1.0 in order to identify the model. In the 1PL case, discrimination parameters were also constrained to be equal across items. Maximum likelihood estimation was employed rather than a Bayesian procedure in order to avoid potential confounds between Bayesian priors used in item calibration and subsequent DIF analysis. Correlations between true and estimate *β*_*j*_ parameters were 0.99 and 1.00 and root mean squared error (RMSE) values were 0.11 and 0.15 for 1PL and 2PL-generated datasets, respectively. For the 2PL data, correlation between true and estimated *α*_*j*_ parameters was .9 and RMSE was 0.14. As previously observed [36], RMSEs for the discrimination parameters increased with higher values of *α*_*j*_. The estimated item parameters were used in subsequent CAT simulations. RMSEs and correlations between item parameters and their estimates were consistent parameter recovery results presented elsewhere [37-39].

#### Generation of CAT Item Response Data

Prior to performing CAT simulations, response data for all 100 items in the simulated item banks described above were generated for a total of 3000 simulees in each iteration. This sample size permitted examination of the effect of CAT item usage on DIF detection rates. Employing the study variables described above, a total of 160 item-response datasets were created and used for CAT simulation. For each dataset, person measures were generated from an *N*(*μ*_CAT_, 1.0) distribution, where μ_CAT_ = 0.0 or 1.0. Non-DIF-item response data were generated using the estimated parameters in Additional file 1. Items simulated to exhibit mode effects (DIF) were randomly selected according to the percentage of DIF items (10% or 30%) for the specified simulation condition. The direction of DIF (i.e., easier vs. more difficult to endorse in the CAT sample) was also randomized. Specifically, a value of 1 (harder to endorse) or −1 (easier to endorse) was generated from a uniform discrete distribution. This value was then multiplied by the appropriate DIF magnitude (0.42 or 0.63 logits), with the resulting value added to the corresponding *β*_*j*_ parameter (see Additional file 1 for table of generated and estimated item parameters and Additional file 2, Additional file 3, Additional file 4 for data files containing these parameters and item response data used in the simulation) The *α*_*j*_ parameters for the generated CAT item responses were the same as those used to generate the initial P&P data.

#### CAT Simulation

Each generated dataset was then used in a series of CAT simulations. In order to ensure comparability across conditions, a fixed-length CAT consisting of 30 administered items for each simulee was conducted. This stopping rule is similar to that used in a previous investigation of CAT and DIF [40]. All CAT simulations employed maximum-likelihood estimation and item selection based on Fisher’s information criterion, a standard CAT algorithm. Each CAT simulation produced the following data: (1) item responses of items selected during the simulated CAT session, (2) index numbers identifying the items selected by CAT, and (3) estimated theta and standard error of theta for each CAT simulee. The originally simulated P&P response data and simulated CAT item-response data were employed in the DIF analysis procedures described earlier (see “A Bayesian Procedure for Detecting Item-Level Mode Effects”).

### Analysis

Prior to addressing the main research questions, descriptive analyses were performed for both the CAT simulation results and the *RZ* and *CrI* statistics. Descriptive statistics for the CAT simulations included CAT-to-full-scale correlations and mean standard errors (MSE), Distributional properties of the *RZ* and *CrI* statistics, including mean, standard deviation, skewness, kurtosis, and values corresponding to the 2.5 and 97.5 percentiles were calculated.

#### Detection of Mode Effects (Research Question 1)

The overall performance of the robust *Z* (*RZ*) and Bayesian credible interval (*CrI,* as measured by the minimum difference of *CrI* to 0 or Δ *CrI*) was assessed first by examining the sensitivity, specificity, and correct classification rates using cutoff values for α = .05 (i.e., | *RZ*| > 1.96 and 95% Δ *CrI* ≠ 0).

Logistic regression and ROC analyses were also performed to examine the predictive accuracy of each statistic without reference to specific cutoff values. Since both *RZ* and Δ *CrI* can have negative and positive values that are indicative of mode DIF, we first fit a logistic regression model with a quadratic term (i.e., *RZ* + *RZ*^2^ and Δ*CrI* + Δ *CrI*^2^ for robust *Z* and credible interval models, respectively) to predict simulated mode DIF. ROC analyses were then conducted based on predicted probabilities from each logistic regression model. The difference in the area under the ROC curves (*AUC*s) was also assessed for statistical significance using a chi-square procedure [41]. Descriptive statistics (percentages) were used to summarize the true positive and false positive mode-of-administration DIF results in the simulation study.

#### Factors Related to True and False Positive Mode Effects (Research Question 2)

A series of multilevel random-intercept logistic regression analyses were performed at both univariate (single predictor) and multivariate levels. At the multivariate level, four models were developed, one for each statistical test (*RZ* and Δ *CrI*) and each DIF decision (correct and incorrect). In each model, the main predictors are: (a) size of DIF, (b) percentage of DIF items in the dataset, (c) IRT model used to generate the response data, (d) difference in mean performance between the P&P and CAT samples (0 vs. 1 logit), (e) number of times a given item was administered by CAT (item usage), (f) item difficulty, and (g) item discrimination, the latter two predictors based on the estimated parameters using the simulated P&P dataset. Preliminary analyses revealed that absolute values of item difficulty better predicted correct DIF detection, whereas signed item difficulty values more accurately predicted incorrect DIF decisions. With the exception of binary variables (i.e., IRT model, difference in CAT vs. P&P mean trait level), predictors were normalized by dividing each variable by two standard deviations prior to analysis [42]. *AUC* values derived from ROC analyses based on each model and each individual were also reported to indicate predictive efficacy. Random intercepts were estimated at both item and dataset levels.

#### Relationship of Item Difficulty to Power and Type I Error

In order to provide a clearer picture of the relationship of item difficulty with power and Type I error, a plot of mean power and Type I error by P&P item difficulty was created. This plot was based on a series of linear regression analyses to predict mean power and Type I error for both *RZ* and *CrI* using the paper-and-pencil item difficulties and their higher level (i.e., quadratic, cubic, quartic, and quintic) terms as predictors. Predicted values from these regression analyses were used to create the plot.

### Software

Generation of item and person parameters and item response data was performed in the R statistical package [43]. Estimation of P&P item parameters was performed using MPlus version 6.0 [44]. CAT simulations were performed with Firestar version 1.33 [45]. For the DIF procedures, estimation in Steps 1 and 2 of the DIF analyses outlined above was performed using WinBUGS version 1.4.3 [46]; see Additional file 5, which has been used in previous IRT applications [28,30,39]. Specifically, we called WinBUGS from R using the R2WinBUGS package [47], the latter used to retrieve the posterior estimates generated by WinBUGS for subsequent analysis. Descriptive analyses and analyses of the simulation results were performed in Stata version 11.0 (Stata Corp., College Station, Texas).

## Results

### Descriptive analyses

#### CAT Simulation

The CAT simulations are summarized in Table 1. As seen in Table 1, CAT to full-instrument correlations were .97 across all conditions. MSEs were 0.26 for the 1PL and 0.23-0.24 for the 2PL conditions. Comparable results were observed as a function of DIF magnitude and percentage and mean *θ*^CAT^.

**Table 1:** Summary of CAT simulations by underlying measurement model, DIF size, mean CAT measures and percentage of DIF items

**IRT Model**	**CAT to Full-Scale *θ *Correlation**				**Mean Standard Error**			
	**1PL**		**2PL**		**1PL**		**2PL**	
**DIF Size**	**0.42**	**0.63**	**0.42**	**0.63**	**0.42**	**0.63**	**0.42**	**0.63**
Diff. Mean *θ*= 0								
DIF % = 10	0.97	0.97	0.97	0.97	0.26	0.26	0.23	0.24
DIF % = 30	0.97	0.96	0.97	0.97	0.26	0.26	0.24	0.24
Diff. Mean *θ*= 1								
DIF % = 10	0.97	0.97	0.97	0.97	0.26	0.26	0.24	0.24
DIF % = 30	0.97	0.96	0.97	0.97	0.26	0.26	0.24	0.24
Average	0.97	0.97	0.97	0.97	0.26	0.26	0.24	0.24

1PL = one-parameter item response model; 2PL = two-parameter item response model; CAT = computerized adaptive testing; DIF = differential item functioning; IRT = item response theory; *θ =* person measure.

With respect to the number of times a given item was administered by CAT (CAT item usage), the median number of item administrations across items and simulation conditions is 553 (*IQR* = 119—1318). The median and *IQR* was 586 (144—1312) and 504 (87—1333) for 1PL and 2PL item banks, respectively. Item usage was comparable for items simulated with DIF (*Med*=557.5, *IQR* = 123—1315) and non-DIF items (*Med* = 551, *IQR* = 117—1320). For *RZ,* an item usage of ≥ 369 and ≥ 422 were associated with power to detect DIF of 80 percent for the 1PL and 2PL conditions, respectively. For *CrI*, 80 percent power was associated with CAT item usage of 305 and 341 for 1PL and 2PL conditions, respectively. In the 2PL condition, item usage was positively correlated with item discrimination (*r =* .46, *p* < .01), reflecting the fact that CAT-bases item selection on item discrimination.

#### Robust Z and 95% Credible Interval Indices

Among non-DIF items, *RZ* had a mean of −0.10 and a standard deviation of 0.82. Mean Δ *CrI* was 0.01 (*SD*=.06). Though both indices were positively skewed and leptokurtotic, this was particularly true for Δ *CrI* (*RZ* skewness = 0.26, Δ *CrI* skewness = 14.94; *RZ* kurtosis = 1.46; Δ *CrI* kurtosis = 253.70). *RZ* values of −1.60 and 1.53 corresponded to the 2.5 and 97.5 percentiles for items not simulated with mode DIF, respectively. Both 2.5^th^ and 97.5^th^ percentiles corresponded to a Δ*CrI* of 0.00 for non-DIF items.

### Detection of mode effects (Research question 1)

Correct classification, sensitivity, and specificity were examined using expected cutoff values at *α* = .05 level, i.e., | *RZ*| > 1.96 and Δ *CrI* ≠ 0. Employing these criteria resulted in correct classification, sensitivity, and specificity of 92.4%, 69.1%, and 98.1% for *RZ* and 92.3%, 71.8%, and 97.2% for Δ *CrI*, respectively. Since our descriptive results presented above suggest that both indices are non-normal, these cutoff values may not be appropriate. We therefore performed logistic regression and ROC analyses to examine the relative performance of the two indices without reference to specific cutoff values. ROC analyses revealed an area under the curve ( *AUC*) of .91 and .82 for *RZ* and Δ *CrI*, respectively. This difference in *AUC*s was statistically significant [ *X*^*2*^(1) = 545.06, *p* < .0001]. This indicates that *RZ* values are significantly stronger predictor of the presence of mode DIF compared to *ΔCrI* values. Further analyses revealed that empirically derived cutoff values for both *RZ* and Δ *CrI* may help to improve sensitivity or specificity. However, since these results are preliminary and for convenience purposes, results presented in subsequent sections of the paper will use the original cutoff values of | *RZ*| > 1.96 and Δ *CrI* ≠ 0.

Table 2 summarizes mean true positive and false positive percentages for each of the simulation conditions. Overall, the false positive rate was well controlled, particularly with *RZ*, with an average false positive rate of 1.9% and ranging from 0.1% to 4.9%. False positive rate was somewhat higher for Δ *CrI*, averaging 2.8% and ranging from 0.1% to 6.9%. The false positive rate for RZ was higher under the large (0.63) DIF effect size condition (*RZ*: 2.3%; Δ *CrI*: 3.1%) relative to the medium (0.42) DIF effect size (*RZ*: 1.5%; Δ *CrI*: 2.4%), and when 30% of the items exhibited DIF (*RZ*: 2.2%; Δ *CrI*: 3.1%) relative to the 10% condition (*RZ*: 1.5%; Δ *CrI*: 2.4%). False positive rates also increased as the difference in mean trait levels between the CAT and P&P modes increased from 0 (*RZ*: 2.8%; Δ *CrI*: 3.0%) to 1.0 logits (*RZ*: 4.4%; Δ *CrI*: 5.8%). The false positive rate increased slightly when data were generated and CAT conducted using the two-parameter IRT model (*RZ*: 2.0%; Δ *CrI*: 2.9%) relative to the 1PL condition (*RZ*: 1.7%; Δ *CrI*: 2.69%). Though these results are promising, it should be noted that 10.2% of datasets evidenced false positive rates above the nominal .05 rate using *RZ* and 15.1% exceeded the 5% false positive threshold when Δ *CrI* was employed.

**Table 2:** True positive and false positive rates as a function of generating IRT model, DIF size, number of DIF items, and mean difference between modes

**IRT Model**	**DIF Size**	**DIF %**	**Diff. Mean *θ***	**Robust *Z***		**Bayes 95% *CrI***	
				**TP%**	**FP%**	**TP%**	**FP%**
1PL	0.42	10	0	54.90	0.91	60.35	1.36
			1	70.00	2.36	66.35	3.88
		30	0	69.66	0.29	70.67	0.44
			1	71.09	1.05	71.91	2.58
	0.63	10	0	78.00	0.56	83.00	0.89
			1	75.00	3.56	81.00	5.22
		30	0	82.67	2.57	87.00	3.00
			1	76.33	2.14	79.33	3.29
2PL	0.42	10	0	60.82	0.06	60.42	0.06
			1	58.24	2.09	55.10	3.89
		30	0	62.71	0.14	66.09	0.28
			1	66.00	4.86	66.33	6.86
	0.63	10	0	72.00	0.33	77.00	0.55
			1	70.00	2.39	77.00	3.56
		30	0	72.33	3.14	77.67	3.14
			1	66.33	3.33	69.00	5.00
Average				69.13	1.86	71.76	2.75

1PL = one-parameter item response model; 2PL = two-parameter item response model; CAT = computerized adaptive testing; DIF = differential item functioning; DIF% = percentage of items simulated with DIF; FP% = percentage of false positive DIF results; IRT = item response theory; TP% = percentage of true positive DIF results; *θ =* person measure.

The present findings revealed power (true positive) rates of 69.1% and 71.8% for *RZ* and Δ *CrI*, respectively. Power was highest in the 1PL condition when DIF was large (0.63 logits) and the percentage of items with DIF was high (30%) and the mean difference in trait level between CAT and P&P modes was 0 (*RZ*: 82.7%; Δ *CrI*: 87.0%). Power was lowest for *RZ* in the 1PL, medium DIF effect size (0.42) 10% DIF items and mean *θ*^CAT^-*θ*^P&P^ = 0 condition (54.9%) whereas for Δ*CrI* it was lowest under the 2PL, medium DIF effect size, 10% DIF items, and mean *θ*^CAT^-*θ*^P&P^ = 1.0 (55.1%). For *RZ*, the average true positive rate was 64.2% when DIF size = 0.42 and 74.1% when DIF size = 0.63 logits. Similarly, true positive rates of 64.7 and 78.9 were observed using Δ *CrI* for medium and large DIF effect sizes, respectively.

### Factors related to true and false positive mode effects (Research question 2)

*W*e examined the relationship of study independent variables, CAT item usage and item parameters on correct (true positive) and incorrect (false positive) DIF decisions by conducting a series of random-intercept multilevel logistic regression analyses, with separate models to predict correct and incorrect DIF decisions based on *RZ* and Δ *CrI*. IRT model used to generate the data and in CAT, DIF size, percentage of DIF, mean difference in trait level, item difficulty, and discrimination (based on values estimated from the simulated P&P data) were used to predict correct and incorrect identification of mode effects and are presented in Tables 3 and 4, respectively. At the univariate level, correct DIF detection was significantly and positively predicted by DIF size, CAT item usage, and item discrimination and significantly and inversely related to the 2PL model and absolute values of P&P item difficulty parameters for both *RZ* and Δ *CrI* statistics. ROC analyses at the univariate level revealed that CAT item usage was most predictive of correct DIF decisions (*AUC*s=0.94 and 0.92 for *RZ* and Δ *CrI*, respectively) followed by absolute item difficulty (*AUC*s=0.85 and 0.83 for *RZ* and Δ *CrI*, respectively). All significant predictors at the univariate level were also significant in the multivariate model. Though not significant at the univariate level, mean difference in mean trait level by mode of 1 logit was significantly and inversely related to true mode DIF detection in the multivariate model. It is noteworthy that both CAT item usage and absolute item difficulty were significant predictors in the multivariate models given that these variables are strongly and negatively correlated (r=−.67), indicating that items of high and low difficulty are administered less frequently by CAT.

**Table 3:** Univariate and multivariate multilevel logistic regression to predict correct detection of mode effects defined by Robust Z and Bayesian 95% credible interval as a function of study variables

**Model/Predictor**	**Univariate**		**Multivariate**	
	***OR***	***AUC***^***a***^	***OR***	**95% *CI***
**Robust Z (Model AUC = 0.95)**				
Size of DIF	1.49**	0.55	3.42**	(2.58-4.54)
Percentage of DIF	1.17	0.52	1.20	(0.89,1.61)
2PL IRT Model^b^	0.76**	0.53	0.47**	(0.35,0.64)
Diff. Mean *θ* = 1.0	0.99	0.50	0.66**	(0.50,0.87)
CAT Item Usage^c^	21133.86**	0.94	3111.68**	(1417.85,6829.03)
Absolute Item Difficulty^d^	0.03**	0.85	0.10**	(0.07,0.14)
Item Discrimination^d^	3.62**	0.60	3.12**	(2.34,4.17)
**Bayesian 95% Credible Interval (Model AUC = 0.93)**				
Size of DIF	1.73**	0.56	3.52**	(2.73,4.53)
Percentage of DIF	1.17	0.52	1.16	(0.89,1.50)
2PL IRT Model^b^	0.74**	0.53	0.50**	(0.39,0.65)
Diff. Mean *θ* = 1.0	0.91	0.49	0.60**	(0.47,0.77)
CAT Item Usage^c^	2468.29**	0.92	505.64**	(264.29,967.37)
Absolute Item Difficulty^d^	0.04**	0.83	0.15**	(0.11,0.20)
Item Discrimination^d^	2.86**	0.58	1.99**	(1.54,2.56)

Correct Detection of Mode Effects = true positive detection of mode DIF among items simulated with mode DIF; AUC = area under the ROC curve; CI = 95% confidence interval; IRT = item response theory model used to generate response data and parameters used in CAT; CAT item usage = number of times a given item was administered by CAT divided by 100; * p < .05; ** p < .01.

**Table 4:** Univariate and multivariate multilevel logistic regression to predict incorrect detection of mode effects defined by Robust Z and Bayesian 95% credible interval as a function of study variables

**Model/Predictor**	**Univariate**		**Multivariate**	
	***OR***	***AUC***^***a***^	***OR***	**95% *CI***
**Robust Z (Model AUC = 0.77)**				
Size of DIF	1.93**	0.55	2.01**	(1.36,2.97)
Percentage of DIF	1.44	0.55	1.48	(0.99,2.20)
2PL IRT Model	1.14	0.52	0.96	(0.63,1.46)
Diff. Mean *θ* = 1.0	3.31**	0.59	3.95**	(2.56,6.08)
CAT Item Usage	1.91**	0.54	4.17**	(3.11,5.60)
Item Difficulty	0.28**	0.62	0.12**	(0.08,0.19)
Item Discrimination	1.64**	0.56	1.23	(0.96,1.58)
**Bayesian 95% Credible Interval (Model AUC = 0.74)**				
Size of DIF	1.62*	0.55	1.61**	(1.20,2.15)
Percentage of DIF	1.33	0.53	1.30	(0.97,1.75)
2PL IRT Model	1.14	0.52	1.08	(0.80,1.47)
Diff. Mean *θ* = 1.0	1.28E+08**	0.62	4.01**	(2.90,5.55)
CAT Item Usage	0.96	0.44	2.36**	(1.82,3.06)
Item Difficulty	0.30**	0.65	0.16**	(0.11,0.22)
Item Discrimination	1.19	0.51	1.02	(0.82,1.26)

Incorrect detection of mode effects = False positive identification of DIF due to mode among items not simulated with mode DIF; AUC = area under the ROC curve; CI = 95% confidence interval; IRT = item response theory model used to generate response data and parameters used in CAT; CAT item usage = number of times a given item was administered by CAT divided by 100; * p < .05; ** p < .01.

For the *RZ* procedure, univariate logistic regression analyses revealed that the following were significantly and positively associated with increased false-positive DIF results: size of DIF, mean difference in mean trait level by mode, CAT item usage, and item discrimination (see Table 4). Conversely, item difficulty was inversely associated with false positive results, indicating that items of higher difficulty were less likely to be incorrectly flagged as exhibiting mode effects. These predictors were also significant at the multivariate level with the exception of item discrimination. For the *CrI* procedure, size of DIF and difference in mean trait level by mode significantly and positively predicted false-positive DIF results, whereas item difficulty was significantly and inversely associated with false positive mode DIF. These factors were also statistically significant in the multivariate model. CAT item usage was also significantly and positively predictive of false positive DIF results in the multivariate model. Based on *AUC*s, item difficulty was the single best predictor of false positives in DIF identification for both *RZ* and *CrI*, followed by difference in mean trait level between modes. The overall model *AUC*s were 0.77 and 0.74 for *RZ* and Δ *CrI* DIF indices, respectively.

#### Relationship of Item Difficulty to Power and Type I Error

**Figure 1:**
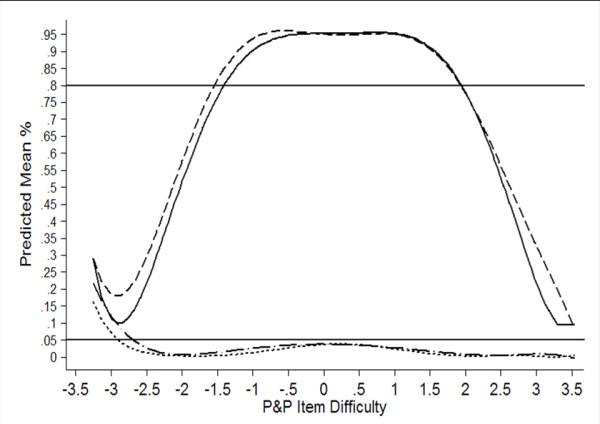
**Mean Predicted True and False Positive Rates by P&P Item Difficulty and Analysis Procedure.** Solid line – Robust z (*RZ*) true positive (TP%) rate. Dashed line – 95% credible interval (*CrI*) true positive rate. Dotted line – Robust Z false positive (FP%) rate. Dash dot line – 95% credible interval false positive rate.

In order to better understand the performance of *RZ* and *CrI* at varying levels of item difficulty, we plotted mean true and false positive rates for both *RZ* and *CrI* as a function of the P&P item difficulty parameters (see Figure 1). This plot reveals that mean false positive rates were well controlled (under the 5% nominal rate) except when item difficulty fell below −2.5 logits. Form −2.5 to −3.5 logits, false positive rate increased from 2% to 15% and from 3% to 20% for *RZ* and *CrI*, respectively. Conversely, true positive rate was ≥ .80 between −1.5 and 2.0 logits for both procedures, though power for *CrI* was slightly higher.

## Discussion

Bayesian methods have been widely used in IRT and have received considerable attention in DIF analysis. However, their application to detecting DIF between CAT and conventional modes of administration has received relatively little attention. Thus, this study sought to develop and test methods for assessing CAT vs. P&P mode DIF employing a Bayesian framework. The present study revealed that the robust *Z* (*RZ*) and Bayesian credible interval (*CrI*) methods generally showed good control of false positive DIF results. Power as measured by the true-positive rate varied considerably for both methods but was consistent with previous reports [25-27]. The *CrI* method resulted in slightly higher power, but this was offset by a higher false positive rate relative to *RZ*. ROC analysis revealed that *RZ* significantly outperformed *CrI*, which appears mainly attributable to improved control of false positives. The results of the study indicate that neither *RZ* nor Δ *CrI* conform to a standard normal or similar distribution. In fact, *RZ* and particularly Δ *CrI* evidenced positive skewness and kurtosis. Thus, empirically derived cutoff values for each statistic may yield improved results. Nevertheless, the use of conventional cutoff values (e.g., 1.96 for *RZ* at α = .05) is not likely to increase Type I error.

CAT item usage was found to be the single best predictor of detecting simulated mode effects, followed by absolute item difficulty. In fact, the multivariate model performed only slightly better than when CAT item usage was the only predictor. For items with DIF, those items administered most often by CAT were more likely to be detected than items administered less frequently. This is not surprising given the wide variability in the frequency that various items were administered during the CAT simulations. The frequency an item is administered by CAT could therefore form the basis of power analysis conducted prior to DIF analysis for a given item. This would be particularly useful in the context of ongoing data collection, potentially improving power and minimizing analysis time.

There are two likely explanations for the observed relationship between absolute item difficulty and power in DIF detection. First, items with difficulty parameters closest to the mean theta values will be more likely to be administered by CAT. Since measures with mean trait levels of 0 or 1 logit were simulated, items in this range of difficulty would be most frequently administered. Second, items towards the extremes of the measurement continuum are less precisely estimated (i.e., have larger standard errors). Thus, power to detect DIF in items that are very easy or difficult to endorse is lower than that for items of average difficulty. This would likely explain why absolute item difficulty was a significant predictor of power even after controlling for CAT item usage. These findings may in part reflect the use of a fixed-length CAT during the simulation. In the case of a variable-length CAT, more items would likely be administered to simulees at the extremes of the trait continuum in order to achieve sufficient measurement precision, including items that are very easy or difficult to endorse. Conversely, we would expect fewer items to be administered to simulees who are in the center of the trait distribution under a variable-length CAT.

With respect to incorrect DIF decisions, easier-to-endorse items were more likely to be erroneously flagged than more difficult items. This finding is in contrast to Wang, Bradlow, Wainer and Muller [31] who found that unlike the standard Mantel-Haenszel test , a Bayesian approach did not result in elevated false positive errors for easy items. There are a number of differences between the Wang, Bradlow, Wainer and Muller study and the present investigation that may account for the differential findings. The former study did not examine DIF in CAT-administered items, employed a testlet model, and analyzed DIF using posterior *p* values. Further, in Wang, Bradlow, Wainer and Muller, Type I error was examined in the absence of DIF items. Conversely, the present study assessed Type I error (false positive DIF results) in which some DIF items were present, thus contaminating the estimated measures used in group matching. Research is clearly needed to determine the causes of elevated false positive rate for easy-to-endorse items. Two possible avenues of research in this area include: (1) further examination of different priors for item parameters and their effect on DIF detection for easy-to-endorse items, and (2) an iterative process of identifying DIF items and then removing or appropriately weighting them in the estimation of person measures.

As might be expected, DIF magnitude (i.e., the difference between CAT and P&P item parameters for a given item) was significantly and positively related to power. The same was not true for the percentage of items with DIF in the item bank. The latter result suggests that the power to detect a single DIF item is not significantly affected by the presence of other DIF items in the bank which may "contaminate" the person measures.

The results of this study revealed a positive relationship between item discrimination and power to identify items with mode DIF. One possible explanation for this finding is that CAT using a 2PL model and maximum information item selection will tend to select items with higher discrimination parameters for administration. In other words, DIF in high discriminating items may be easier to detect because these items are administered more frequently in CAT. Yet the results of the multivariate logistic regression analysis failed to support this conclusion. Item discrimination remained statistically significant even when controlling for CAT item usage. High item discrimination therefore appears to enhance power in mode-effect detection. This finding is corroborated by previous DIF research examining the relationship of item discrimination to power using several analytic procedures [48,49]. Using the *RZ* procedure, item discrimination was positively associated with false DIF results at the univariate level, though this effect was no longer significant at the multivariate level. The latter findings partially confirmed previous studies that reported a positive relationship between item discrimination and Type I error rate for uniform DIF [50,51].

For both *RZ* and *CrI*, power to detect DIF was lower in the 2PL condition. This appears to be related to some extent to CAT item usage. Though the number of items administered to each simulee was the same across the two conditions, median CAT item usage was lower (*Med*=504) in the 2PL than in the 1PL (*Med*=586) condition. However, the logistic regression results indicate that IRT model remained significant even when CAT item usage was included in the model. Thus, CAT item usage may not completely explain why power was lower in the 2PL condition. Though these findings are based on a small number of replications per condition and need to be interpreted cautiously, the observed relationship between measurement model and power to detect mode effects warrants further exploration.

In addition to the effect of item parameters, false positive DIF results were significantly associated with DIF size and mean difference in trait level between CAT and P&P administration modes. These effects likely reflect problems with the trait estimate used as the matching variable in the DIF analysis. Items with large DIF effects and mean differences in trait level between groups limit the effectiveness of matching, as has been observed in previous DIF studies [50-53]. These results highlight the need for careful sampling of respondents who complete each form of the instrument and assessment of trait-level differences prior to assessment of mode effects. The percentage of DIF items in the item bank was not associated with false DIF results. Though false positive rates were smaller in the 10% compared to the 30% DIF conditions, DIF percentage was not found to be significantly predictive of false positive DIF in either the univariate or multivariate logistic regression models for either *RZ* or *CrI*. Note that due to the computational demands involved in estimating posterior distributions of parameters, we decided not to perform item purification in this simulation.

The strength of Monte Carlo simulation lies in its ability to systematically vary several factors thought to affect identification of simulated effects. In this study, several factors were directly examined with respect to detection of mode-of-administration DIF, including DIF size, percentage of DIF items, and mean difference in trait level between modes, item response model, and analytic procedure. We also examined the effects of variables not part of the research design, including CAT item usage, item discrimination, and item difficulty parameters. A particular strength of the study is the examination of CAT item usage rather than sample size as a factor related to identification of DIF.

Nevertheless, our study has several limits. For example, several other factors were not considered in the simulation. Of particular importance is the degree to which the mean, variance, and shape of distributions of parameters are consistent with specified priors in the Bayesian estimation model. Though differences in mean trait levels were examined, deviations from prior assumptions concerning parameter variances or distribution types were not examined. For instance, there is a need to conduct further studies examining the potential effect of skewed theta and item parameter distributions on the performance of DIF procedures [24]. Methods of CAT item selection and stopping rules also deserve further attention. There is also a need to assess the *RZ* and *CrI* procedures in identifying items exhibiting non-uniform mode DIF. Additional limitations of the present study include the small number of replications per experimental condition, the use of a fixed-length CAT and fixed item bank size.

Also, we intentionally did not address non-uniform DIF. Thus limits our study to conclusions about uniform DIF only. Importantly, though, no theoretical reasons exist to preclude conducting similar analyses on non-uniform DIF. However, given the nascent status of research in this field, we choose to focus on a single type of DIF. Our future research will hopefully address non-uniform DIF in one study and both simultaneously in a final study. By addressing each in a stepwise and piecemeal fashion, we hope to avoid spurious conclusions that could arise by addressing all simultaneously in the initial study. For example, we did not want to the presence of non-uniform to influence the detection of uniform DIF using these methods we developed here. Final, we only used simulated data. Future studies employing these procedures with real data are also needed.

## Conclusions

This study yielded mixed results concerning the methods for assessing mode effects. Whereas Type I error was well controlled, power to detect DIF was suboptimal, though the present findings were consistent with those reported in similar studies [25-27]. The modified robust Z test provided better control of the Type I error rate compared to *CrI*. True positive rates were primarily predicted by CAT item usage, absolute item difficulty and item discrimination. Further research is needed to examine the robustness of the method under varying prior assumptions concerning the distribution of item and person parameters and when data fail to conform to these prior assumptions. False identification of DIF when items were very easy to endorse is a problem requiring additional investigation.

## Abbreviation

1PL: One-parameter logistic item response theory model; 2PL: Two-parameter logistic item response theory model; AUC: Area under the curve; CAT: Computerized adaptive testing; CrI: Credible interval; DIF: Differential item functioning; EB: Empirical Bayes; IQR: Interquartile range; IRT: Item response theory; MH: Mantel-Haenszel test; P&P: Paper-and-pencil (administration); RZ: Robust z test.

## Competing interests

The authors declare that they have no competing interests.

## Authors’ contributions

BR conceived of the study, developed the procedures tested in the study, wrote the code for the simulation and performed the statistical analyses. BR and AC participated in the writing of the manuscript. BR wrote the background and methods sections, BR and AC wrote the results, discussion and conclusions sections. Both BR and AC reviewed drafts of the manuscript and gave final approval of the submitted version.

## Pre-publication history

The pre-publication history for this paper can be accessed here:

http://www.biomedcentral.com/1471-2288/12/124/prepub

## Supplementary Material

Additional file 1 **Appendix A.** Generating and Estimated Item Parameters for the Two Simulated Item Banks (Validation data) Based on the One-and Two-Parameter IRT Models, Respectively. 1PL = one-parameter item response model; 2PL = two-parameter item response model; *α* = simulated discrimination parameter; *β* = simulated difficulty parameter; = estimated discrimination parameter; $\hat{\beta }$= estimated difficulty parameter. Click here for file

Additional file 2 **Sample File of Simulee Measures Used to Generate CAT Item Responses.** A 1 column x 3000 row of generated θ values used to generate item responses for subsequent use in CAT simulation. Click here for file

Additional file 3 **Sample P&P and CAT Item Parameters.** This file contains 4 columns of item parameters for 100 items. Columns 1 and 2 are the estimated discrimination and difficulty parameters for the P&P version of the instrument, respectively. Columns 3 and 4 are the discrimination and difficulty parameters used to generate the item responses for subsequent use in the CAT simulation. Click here for file

Additional file 4 **Sample Item Response File Used in CAT Simulation.** A 100 column by 3000 row spreadsheet containing generated item responses according to the CAT person and item parameters described above. A 1 indicates a "no" response and 2 a "yes" response. Click here for file

Additional file 5 **Appendix B.** WinBUGS Code. Click here for file
