# Supplementary material for: Comparison of two Bayesian methods to detect mode effects between paper-based and computerized adaptive assessments: a preliminary Monte Carlo study
Source: BMC Med Res Methodol. 2012 Aug 17;12:124. doi: 10.1186/1471-2288-12-124 (PMC3552735; doi:10.1186/1471-2288-12-124)
Supplement: Additional file 1 — Appendix A. Generating and Estimated Item Parameters for the Two Simulated Item Banks (Validation data) Based on the One-and Two-Parameter IRT Models, Respectively. 1PL = one-parameter item response model; 2PL = two-parameter item response model; α = simulated discrimination parameter; β = simulated difficulty parameter; = estimated discrimination parameter; β^= estimated difficulty parameter. [file 1471-2288-12-124-S1.doc]

| **Item** | **Item Bank 1 (I PL)** | | | | **Item Bank 2 (2PL)** | | | |
| --- | --- | --- | --- | --- | --- | --- | --- | --- |
|  | *α* | ***Β*** |  | $\hat{\text{β}}$ | ***α*** | ***β*** | $\hat{\text{α}}$ | $\hat{\text{β}}$ |
| 1 | 1.000 | -3.000 | 0.986 | -3.254 | 1.230 | -3.000 | 1.072 | -3.151 |
| 2 | 1.000 | -3.000 | 0.986 | -2.988 | 0.940 | -3.000 | 0.758 | -3.137 |
| 3 | 1.000 | -3.000 | 0.986 | -3.128 | 0.875 | -3.000 | 0.900 | -2.856 |
| 4 | 1.000 | -3.000 | 0.986 | -2.746 | 0.975 | -3.000 | 1.042 | -2.816 |
| 5 | 1.000 | -2.750 | 0.986 | -2.581 | 1.178 | -2.750 | 1.141 | -2.765 |
| 6 | 1.000 | -2.750 | 0.986 | -2.819 | 1.413 | -2.750 | 1.871 | -2.353 |
| 7 | 1.000 | -2.750 | 0.986 | -2.620 | 0.893 | -2.750 | 0.823 | -2.904 |
| 8 | 1.000 | -2.750 | 0.986 | -2.581 | 1.402 | -2.750 | 1.238 | -3.014 |
| 9 | 1.000 | -2.500 | 0.986 | -2.384 | 1.395 | -2.500 | 1.370 | -2.595 |
| 10 | 1.000 | -2.500 | 0.986 | -2.680 | 0.955 | -2.500 | 1.001 | -2.430 |
| 11 | 1.000 | -2.500 | 0.986 | -2.581 | 1.680 | -2.500 | 1.488 | -2.567 |
| 12 | 1.000 | -2.500 | 0.986 | -2.384 | 1.010 | -2.500 | 1.101 | -2.440 |
| 13 | 1.000 | -2.250 | 0.986 | -2.248 | 0.721 | -2.250 | 0.737 | -2.187 |
| 14 | 1.000 | -2.250 | 0.986 | -2.287 | 0.902 | -2.250 | 0.802 | -2.272 |
| 15 | 1.000 | -2.250 | 0.986 | -2.109 | 1.120 | -2.250 | 1.075 | -2.256 |
| 16 | 1.000 | -2.250 | 0.986 | -2.224 | 1.236 | -2.250 | 1.274 | -2.138 |
| 17 | 1.000 | -2.000 | 0.986 | -2.046 | 0.766 | -2.000 | 0.930 | -1.561 |
| 18 | 1.000 | -2.000 | 0.986 | -2.016 | 0.997 | -2.000 | 0.980 | -2.026 |
| 19 | 1.000 | -2.000 | 0.986 | -2.067 | 1.049 | -2.000 | 1.034 | -1.972 |
| 20 | 1.000 | -2.000 | 0.986 | -1.997 | 1.190 | -2.000 | 1.143 | -1.955 |
| 21 | 1.000 | -1.750 | 0.986 | -1.742 | 0.844 | -1.750 | 0.858 | -1.709 |
| 22 | 1.000 | -1.750 | 0.986 | -1.661 | 0.742 | -1.750 | 0.781 | -1.638 |
| 23 | 1.000 | -1.750 | 0.986 | -1.705 | 0.967 | -1.750 | 1.076 | -1.702 |
| 24 | 1.000 | -1.750 | 0.986 | -1.734 | 1.151 | -1.750 | 1.040 | -1.677 |
| 25 | 1.000 | -1.500 | 0.986 | -1.383 | 0.824 | -1.500 | 0.896 | -1.417 |
| 26 | 1.000 | -1.500 | 0.986 | -1.448 | 0.979 | -1.500 | 0.801 | -1.599 |
| 27 | 1.000 | -1.500 | 0.986 | -1.528 | 1.008 | -1.500 | 1.050 | -1.435 |
| 28 | 1.000 | -1.500 | 0.986 | -1.412 | 0.970 | -1.500 | 0.821 | -1.512 |
| 29 | 1.000 | -1.250 | 0.986 | -1.190 | 0.955 | -1.250 | 0.894 | -1.239 |
| 30 | 1.000 | -1.250 | 0.986 | -1.111 | 0.767 | -1.250 | 0.760 | -1.150 |
| 31 | 1.000 | -1.250 | 0.986 | -1.236 | 1.333 | -1.250 | 1.301 | -1.183 |
| 32 | 1.000 | -1.250 | 0.986 | -1.221 | 0.774 | -1.250 | 0.777 | -1.183 |
| 33 | 1.000 | -1.000 | 0.986 | -0.963 | 1.287 | -1.000 | 1.285 | -0.908 |
| 34 | 1.000 | -1.000 | 0.986 | -0.981 | 1.058 | -1.000 | 0.949 | -1.012 |
| 35 | 1.000 | -1.000 | 0.986 | -0.976 | 1.003 | -1.000 | 1.044 | -0.990 |
| 36 | 1.000 | -1.000 | 0.986 | -0.816 | 0.810 | -1.000 | 0.914 | -0.826 |
| 37 | 1.000 | -0.750 | 0.986 | -0.721 | 0.766 | -0.750 | 0.760 | -0.649 |
| 38 | 1.000 | -0.750 | 0.986 | -0.738 | 1.359 | -0.750 | 1.401 | -0.629 |
| 39 | 1.000 | -0.750 | 0.986 | -0.730 | 0.961 | -0.750 | 0.981 | -0.680 |
| 40 | 1.000 | -0.750 | 0.986 | -0.725 | 1.402 | -0.750 | 1.547 | -0.581 |
| 41 | 1.000 | -0.500 | 0.986 | -0.491 | 1.119 | -0.500 | 1.044 | -0.377 |
| 42 | 1.000 | -0.500 | 0.986 | -0.457 | 2.000 | -0.500 | 1.778 | -0.381 |
| 43 | 1.000 | -0.500 | 0.986 | -0.457 | 0.860 | -0.500 | 0.753 | -0.468 |
| 44 | 1.000 | -0.500 | 0.986 | -0.506 | 1.018 | -0.500 | 0.905 | -0.543 |
| 45 | 1.000 | -0.250 | 0.986 | -0.244 | 1.022 | -0.250 | 1.127 | -0.161 |
| 46 | 1.000 | -0.250 | 0.986 | -0.193 | 1.059 | -0.250 | 1.079 | -0.171 |
| 47 | 1.000 | -0.250 | 0.986 | -0.067 | 0.905 | -0.250 | 0.770 | -0.199 |
| 48 | 1.000 | -0.250 | 0.986 | -0.266 | 1.262 | -0.250 | 1.347 | -0.102 |
| 49 | 1.000 | 0.000 | 0.986 | 0.048 | 0.836 | 0.000 | 0.695 | 0.025 |
| 50 | 1.000 | 0.000 | 0.986 | 0.080 | 0.839 | 0.000 | 0.715 | 0.055 |
| 51 | 1.000 | 0.000 | 0.986 | 0.015 | 0.523 | 0.000 | 0.532 | 0.085 |
| 52 | 1.000 | 0.000 | 0.986 | 0.026 | 1.116 | 0.000 | 1.015 | 0.192 |
| 53 | 1.000 | 0.250 | 0.986 | 0.339 | 0.793 | 0.250 | 0.831 | 0.326 |
| 54 | 1.000 | 0.250 | 0.986 | 0.331 | 1.619 | 0.250 | 1.765 | 0.297 |
| 55 | 1.000 | 0.250 | 0.986 | 0.306 | 1.735 | 0.250 | 1.621 | 0.333 |
| 56 | 1.000 | 0.250 | 0.986 | 0.306 | 0.804 | 0.250 | 0.783 | 0.272 |
| 57 | 1.000 | 0.500 | 0.986 | 0.482 | 0.866 | 0.500 | 0.829 | 0.673 |
| 58 | 1.000 | 0.500 | 0.986 | 0.583 | 1.008 | 0.500 | 1.048 | 0.602 |
| 59 | 1.000 | 0.500 | 0.986 | 0.615 | 0.914 | 0.500 | 0.898 | 0.554 |
| 60 | 1.000 | 0.500 | 0.986 | 0.615 | 1.304 | 0.500 | 1.276 | 0.673 |
| 61 | 1.000 | 0.750 | 0.986 | 0.805 | 1.640 | 0.750 | 1.737 | 0.886 |
| 62 | 1.000 | 0.750 | 0.986 | 0.835 | 1.062 | 0.750 | 1.014 | 0.761 |
| 63 | 1.000 | 0.750 | 0.986 | 0.848 | 1.077 | 0.750 | 1.002 | 0.894 |
| 64 | 1.000 | 0.750 | 0.986 | 0.839 | 1.175 | 0.750 | 1.278 | 0.856 |
| 65 | 1.000 | 1.000 | 0.986 | 1.054 | 1.289 | 1.000 | 1.248 | 1.111 |
| 66 | 1.000 | 1.000 | 0.986 | 1.128 | 1.062 | 1.000 | 0.937 | 1.145 |
| 67 | 1.000 | 1.000 | 0.986 | 1.030 | 0.790 | 1.000 | 0.765 | 1.105 |
| 68 | 1.000 | 1.000 | 0.986 | 1.030 | 1.360 | 1.000 | 1.393 | 1.102 |
| 69 | 1.000 | 1.250 | 0.986 | 1.431 | 1.504 | 1.250 | 1.488 | 1.314 |
| 70 | 1.000 | 1.250 | 0.986 | 1.450 | 0.648 | 1.250 | 0.568 | 1.673 |
| 71 | 1.000 | 1.250 | 0.986 | 1.425 | 1.084 | 1.250 | 0.970 | 1.390 |
| 72 | 1.000 | 1.250 | 0.986 | 1.291 | 1.289 | 1.250 | 1.274 | 1.482 |
| 73 | 1.000 | 1.500 | 0.986 | 1.566 | 0.775 | 1.500 | 0.741 | 1.636 |
| 74 | 1.000 | 1.500 | 0.986 | 1.539 | 0.685 | 1.500 | 0.619 | 1.617 |
| 75 | 1.000 | 1.500 | 0.986 | 1.519 | 1.253 | 1.500 | 1.101 | 1.580 |
| 76 | 1.000 | 1.500 | 0.986 | 1.586 | 0.920 | 1.500 | 0.960 | 1.587 |
| 77 | 1.000 | 1.750 | 0.986 | 1.779 | 1.773 | 1.750 | 1.900 | 1.787 |
| 78 | 1.000 | 1.750 | 0.986 | 1.755 | 0.890 | 1.750 | 0.987 | 1.776 |
| 79 | 1.000 | 1.750 | 0.986 | 1.829 | 0.934 | 1.750 | 0.755 | 2.018 |
| 80 | 1.000 | 1.750 | 0.986 | 1.963 | 1.553 | 1.750 | 1.816 | 1.807 |
| 81 | 1.000 | 2.000 | 0.986 | 2.094 | 0.534 | 2.000 | 0.550 | 1.952 |
| 82 | 1.000 | 2.000 | 0.986 | 2.073 | 0.901 | 2.000 | 0.804 | 2.191 |
| 83 | 1.000 | 2.000 | 0.986 | 2.052 | 1.416 | 2.000 | 1.385 | 2.143 |
| 84 | 1.000 | 2.000 | 0.986 | 1.972 | 1.359 | 2.000 | 1.351 | 2.024 |
| 85 | 1.000 | 2.250 | 0.986 | 2.353 | 1.142 | 2.250 | 1.069 | 2.490 |
| 86 | 1.000 | 2.250 | 0.986 | 2.490 | 1.217 | 2.250 | 1.206 | 2.278 |
| 87 | 1.000 | 2.250 | 0.986 | 2.381 | 1.207 | 2.250 | 1.038 | 2.410 |
| 88 | 1.000 | 2.250 | 0.986 | 2.381 | 1.276 | 2.250 | 1.524 | 2.204 |
| 89 | 1.000 | 2.500 | 0.986 | 2.541 | 1.632 | 2.500 | 1.534 | 2.546 |
| 90 | 1.000 | 2.500 | 0.986 | 2.633 | 0.951 | 2.500 | 0.989 | 2.520 |
| 91 | 1.000 | 2.500 | 0.986 | 2.806 | 1.053 | 2.500 | 1.462 | 2.174 |
| 92 | 1.000 | 2.500 | 0.986 | 2.633 | 1.345 | 2.500 | 1.548 | 2.463 |
| 93 | 1.000 | 2.750 | 0.986 | 2.831 | 1.250 | 2.750 | 1.076 | 3.018 |
| 94 | 1.000 | 2.750 | 0.986 | 3.027 | 1.100 | 2.750 | 1.219 | 2.621 |
| 95 | 1.000 | 2.750 | 0.986 | 2.909 | 0.860 | 2.750 | 0.966 | 2.552 |
| 96 | 1.000 | 2.750 | 0.986 | 2.937 | 1.060 | 2.750 | 1.071 | 2.665 |
| 97 | 1.000 | 3.000 | 0.986 | 3.027 | 1.126 | 3.000 | 1.378 | 2.973 |
| 98 | 1.000 | 3.000 | 0.986 | 3.059 | 0.928 | 3.000 | 0.718 | 3.537 |
| 99 | 1.000 | 3.000 | 0.986 | 3.291 | 1.102 | 3.000 | 1.757 | 2.626 |
| 100 | 1.000 | 3.000 | 0.986 | 3.129 | 0.649 | 3.000 | 0.712 | 3.118 |
| *Mean* | 1.000 | 0.000 | 0.986 | 0.066 | 1.084 | 0.000 | 1.084 | 0.066 |
