# Supplementary material for: Comparison of two Bayesian methods to detect mode effects between paper-based and computerized adaptive assessments: a preliminary Monte Carlo study
Source: BMC Med Res Methodol. 2012 Aug 17;12:124. doi: 10.1186/1471-2288-12-124 (PMC3552735; doi:10.1186/1471-2288-12-124)
Supplement: Additional file 5 — Appendix B. WinBUGS Code. [file 1471-2288-12-124-S5.doc]

**Appendix B: WinBUGS Code**

On the next page is the WinBUGS code used to generate the posterior distribution of person and item parameters. Three sets of item responses (for P&P, CAT, and combined modes, named resp1, resp2, and resp3, respectively) are passed to WinBUGS via the R2WinBUGS function. Also passed to WinBUGS are the variables L (number of items) and N1, N2, and N3 representing the number of cases in each response set. The parameters based on the combined (CAT and P&P) item responses are estimated in lines 2-8. Item parameters for the P&P data are estimated in lines 9-15 and the CAT parameters in lines 16-22. Priors for the P&P and CAT item parameters are specified in lines 23-32. Note that priors for person and item parameters are specified in the form *distribution*(mean, precision), where *distribution* is either normal (dnorm) or lognormal (dlnorm) and precision is equal to 1/σ^2^. In addition, priors for person measures estimated from the P&P (theta1[]) and CAT (theta2[]) are not specified, because these parameters are not estimated. Rather, the values for theta1[] and theta2[] are taken directly from theta[], the person measures estimated from the combined data. The use of the cut() function (see lines 10 and 17) “cuts” feedback to (i.e., prevents modification of) theta[] resulting from estimation of P&P- and CAT-specific parameters. This effectively ensures that mode-specific item parameters are estimated based on an anchored set of person measures based on the combined response data.

WinBUGS Code

| 1 | model { |
| --- | --- |
| 2 | for ( i in 1 : N3 ) { |
| 3 | for (j in 1 : L ) { |
| 4 | *p*[i,j] <- exp(1.702 * a[j] * (theta[i]-diff[j])) / (1 + exp(1.702 * a[j] * (theta[i]-diff[j]))) |
| 5 | resp3[i,j] ~ dbern(*p*[i,j]) |
| 6 | } |
| 7 | theta[i] ~ dnorm(0,1) |
| 8 | } |
|  |  |
| 9 | for ( i in 1 : N1 ) { |
| 10 | theta1[i] <- cut(theta[i]) |
| 11 | for (j in 1 : L ) { |
| 12 | p1[i,j] <- exp(1.702* a1[j] * (theta1[i] - diff1[j])) / (1 + exp(1.702 * a1[j] *(theta1[i] - diff1[j]))) |
| 13 | resp1[i,j] ~ dbern(p1[i,j]) |
| 14 | } |
| 15 | } |
| 16 | for ( i in 1 : N2 ) { |
| 17 | theta2[i] <- cut(theta[i + N1]) |
| 18 | for (j in 1 : L ) { |
| 19 | p2[i,j] <- exp(1.702 * a2[j] * (theta2[i] - diff2[j])) / (1 + exp(1.702 * a2[j] * (theta2[i] - diff2[j]))) |
| 20 | resp2[i,j] ~ dbern(p2[i,j]) |
| 21 | } |
| 22 | } |
| 23 | for (j in 1 : L) { |
| 24 | diff1[j] ~ dnorm(0,0.5) |
| 25 | a1[j] ~ dlnorm(0,2) |
| 26 | diff2[j] ~ dnorm(0,0.5) |
| 27 | a2[j] ~ dlnorm(0,2) |
| 28 | } |
|  |  |
| 29 | for (j in 1 : L ) { |
| 30 | diff[j] ~ dnorm(0,0.5) |
| 31 | a[j] ~ dlnorm(0,2) |
| 32 | } |
| 33 | } |
